# Genome-Wide Identification and Characterization of *SOD*s in Zhikong Scallop Reveals Gene Expansion and Regulation Divergence after Toxic Dinoflagellate Exposure

**DOI:** 10.3390/md17120700

**Published:** 2019-12-12

**Authors:** Shanshan Lian, Liang Zhao, Xiaogang Xun, Jiarun Lou, Moli Li, Xu Li, Shi Wang, Lingling Zhang, Xiaoli Hu, Zhenmin Bao

**Affiliations:** 1 MOE Key Laboratory of Marine Genetics and Breeding, Ocean University of China, Qingdao 266003, China; lianshanshan@ouc.edu.cn (S.L.); ouczhaoliang@126.com (L.Z.); xxgsemail@126.com (X.X.); ljr9481@163.com (J.L.); lml@stu.ouc.edu.cn (M.L.); 13655321920@163.com (X.L.); swang@ouc.edu.cn (S.W.); lingling80@ouc.edu.cn (L.Z.); zmbao@ouc.edu.cn (Z.B.); 2 Laboratory for Marine Fisheries Science and Food Production Processes, Qingdao National Laboratory for Marine Science and Technology, Qingdao 266237, China; 3 Laboratory for Marine Biology and Biotechnology, Qingdao National Laboratory for Marine Science and Technology, Qingdao 266237, China

**Keywords:** *SOD* family, bivalves, PST challenge, adaptation, genomic analysis, expression profiling

## Abstract

As filter-feeding animals mainly ingesting microalgae, bivalves could accumulate paralytic shellfish toxins (PSTs) produced by harmful algae through diet. To protect themselves from the toxic effects of PSTs, especially the concomitant oxidative damage, the production of superoxide dismutase (SOD), which is the only eukaryotic metalloenzyme capable of detoxifying superoxide, may assist with toxin tolerance in bivalves. To better understand this process, in the present study, we performed the first systematic analysis of *SOD* genes in bivalve *Chlamys farreri*, an important aquaculture species in China. A total of six *Cu/Zn-SOD*s (*SOD1-6*) and two *Mn-SOD*s (*SOD7*, *SOD8*) were identified in *C. farreri*, with gene expansion being revealed in *Cu/Zn-SOD*s. In scallops exposed to two different PSTs-producing dinoflagellates, *Alexandrium minutum* and *A. catenella*, expression regulation of *SOD* genes was analyzed in the top ranked toxin-rich organs, the hepatopancreas and the kidney. In hepatopancreas, which mainly accumulates the incoming PSTs, all of the six *Cu/Zn-SOD*s showed significant alterations after *A. minutum* exposure, with *SOD1*, *2*, *3*, *5*, and *6* being up-regulated, and *SOD4* being down-regulated, while no significant change was detected in *Mn-SOD*s. After *A. catenella* exposure, up-regulation was observed in *SOD2*, *4*, *6*, and *8*, and *SOD7* was down-regulated. In the kidney, where PSTs transformation occurs, *SOD4*, *5*, *6*, and *8* were up-regulated, and *SOD7* was down-regulated in response to *A. minutum* feeding. After *A. catenella* exposure, all the *Cu/Zn-SOD*s except *SOD1* were up-regulated, and *SOD7* was down-regulated in kidney. Overall, in scallops after ingesting different toxic algae, *SOD* up-regulation mainly occurred in the expanded *Cu/Zn-SOD* group, and *SOD6* was the only member being up-regulated in both toxic organs, which also showed the highest fold change among all the *SOD*s, implying the importance of *SOD6* in protecting scallops from the stress of PSTs. Our results suggest the diverse function of scallop *SOD*s in response to the PST-producing algae challenge, and the expansion of *Cu/Zn-SOD*s might be implicated in the adaptive evolution of scallops or bivalves with respect to antioxidant defense against the ingested toxic algae.

## 1. Introduction

Reactive oxygen species (ROS) are the active partially excited forms of oxygen that are usually unavoidably generated as by-products of aerobic metabolism and can damage multiple cellular components [1]. Superoxide dismutase (SOD) (EC 1.15.1.1) is the only eukaryotic metalloenzyme capable of detoxifying superoxide, one type of ROS, principally acting as the first and most important line of antioxidant defense among all eukaryotic organisms [1,2]. Based on distinct catalytic metal cofactors, superoxide dismutases have been classified as copper/zinc SODs and manganese SODs in eukaryotic animals [3]. Usually, Mn-SODs are considered as an early evolutionary form of SODs, since they were found mainly in mitochondria, the organelle which is thought have originally been independent organisms and were symbiotically fused into eukaryotes one billion years ago [4]. Cu/Zn-SODs reside in both the extracellular milieu and numerous intracellular compartments, such as the cytoplasm, lysosomes, peroxisomes, and nuclei, and are likely to be the most recently evolved forms [5]. Multiple lines of evidence have shown that *SOD* genes are sensitive to stress [2,6], and altered *SOD* regulation has been implicated in a broad range of physically deteriorative states, including abnormal development [7], neurodegeneration [8,9,10], immunosuppression [11,12], and neoplasm formation [13,14]. Previous studies also revealed that *SOD* genes were involved in response to toxic challenge. For instance, in zebrafish, researchers found that low concentrations of toxic HgCl_2_ exposure could significantly induce the expression of *Cu/Zn-SOD* in muscle, with dose-dependent enhancement of SOD activity [15]. In muscle afferent DRG (dorsal root ganglia) neurons of rats, a lower superoxide level owing to the addition of SOD could modulate the current of voltage-gated sodium channels (Na_v_), the main target site under tetrodotoxin stress [16].

Bivalve mollusk, as descendants of an early Cambrian lineage that appeared over 500 million years ago, are well adapted to benthic filter feeding and thrive in highly dynamic oceans and freshwater environments with a variety of stressors. *SOD*s are reported to be inducible in bivalves through biotic and/or abiotic factors, such as toxicants [17], hypoxia [18], and pathogenic organisms [19], and *SOD*s could appear in response to large fluctuation in water temperature and salinity [20,21]. As natural filter feeders on algae, bivalve mollusk can accumulate toxins produced by the ingested algae, especially during harmful algal blooms [22,23]. Paralytic shellfish toxins (PSTs) mainly produced by dinoflagellates are among the most potent marine biotoxins [24,25], which can bind to the Na_v_, causing the blockage of neuronal activity [22,26]. Although bivalves can tolerate much higher concentrations of PSTs than human and other mammals, largely due to possessing toxin-resistant amino acids in Na_v_, PSTs accumulation causes changes in the behaviors and metabolism processes of bivalves [27,28,29]. ROS production and alternation of SOD activity occurred in PST-contaminated bivalve [28,30], making antioxidant defense a sine qua non for coping with PSTs. For example, Cao et al. documented that, under saxitoxin (STX, the most potent PST) exposure, SOD activity was significantly increased in oyster *Crassostrea gigas* and scallop *Chlamys farreri* [31]. SOD activity was also found to be induced in muscle and hepatopancreas of mussels (*Mytilus galloprovincialis*) fed with toxic *Alexandrium tamarense* [32]. However, the involvement of bivalve SODs in response to PST producing algae might be species-or tissue-specific, as no change of SOD activity was observed in mantle and hepatopancreas of scallop *Nodipecten subnodosus* exposed to *Gymnodinium catenatum* but decreased in gill and muscle [33]. Therefore, more research is needed to explore the functions of SODs and antioxidant system in bivalves. Considering that SOD activity was exerted by the proteins expressed by several *SOD* genes, the systematic analysis of *SOD*s in bivalve genomes and their expression regulation during PSTs producing algae exposure, could increase our understanding of the roles of *SOD*s in antioxidant defense against toxic algae, as related studies are currently very limited.

Compared with many other bivalve species, the scallop could accumulate PSTs with higher concentrations and longer retention times [34], making it an ideal species for research on toxin stress and tolerance in bivalves. In the present study, we performed systematic identification of *SOD* genes in Zhikong scallop, *C. farreri* (Jones et Preston, 1904), a Chinese native species which has been repeatedly reported for PSTs contamination [25,31]. We also analyzed the spatiotemporal expression patterns of all the *SOD* family members, and their transcriptional responses to the exposure of PST producing algae, *A. minutum* (strain AM-1) and *A. catenella* (strain ACDH), in the two most toxic organs, hepatopancreas and kidney of *C. farreri*. To the best of our knowledge, this is the first comprehensive study of the *SOD* gene family in bivalves, notably providing valuable information regarding the classification, evolution, and putative functions of these genes. Additionally, the diverse expression regulation of scallop *SOD*s in response to toxic algae exposure between organs and between algae species will improve our understanding of PST-induced antioxidant responses and protection in bivalves.

## 2. Results and Discussion

### 2.1. Identification of SOD Genes in C. farreri

In mammals, only three unique and highly compartmentalized *SOD*s have been identified. By contrast, a total of eight *SOD*s, including six *Cu/Zn-SOD*s (*SOD1-6*) and two *Mn-SOD*s (*SOD7, 8*), have been found in the *C. farreri* genome, revealing the expansion of *Cu/Zn-SOD*. To further test whether other marine animals showed expansion phenomena of the *SOD* family, we also retrieved *SOD* genes in oyster *C. gigas*, snail *Biomphalaria glabrata,* and sea urchin *Strongylocentrotus purpuratus*, and our results showed that more than six *SOD* members were present in these species. It is noteworthy that, just as in *C. farreri*, most *SOD* expansion events in these marine animals occurred within the Cu/Zn group. Among *Cu/Zn-SOD*s, *SOD2*, and *SOD3* were found to be located on the same chromosome in *C. farreri*, indicating that tandem gene duplication may occur. The two *Mn-SOD*s in *C. farreri* were found respectively localized on chromosomes 2 and 19.

**Figure 1:**
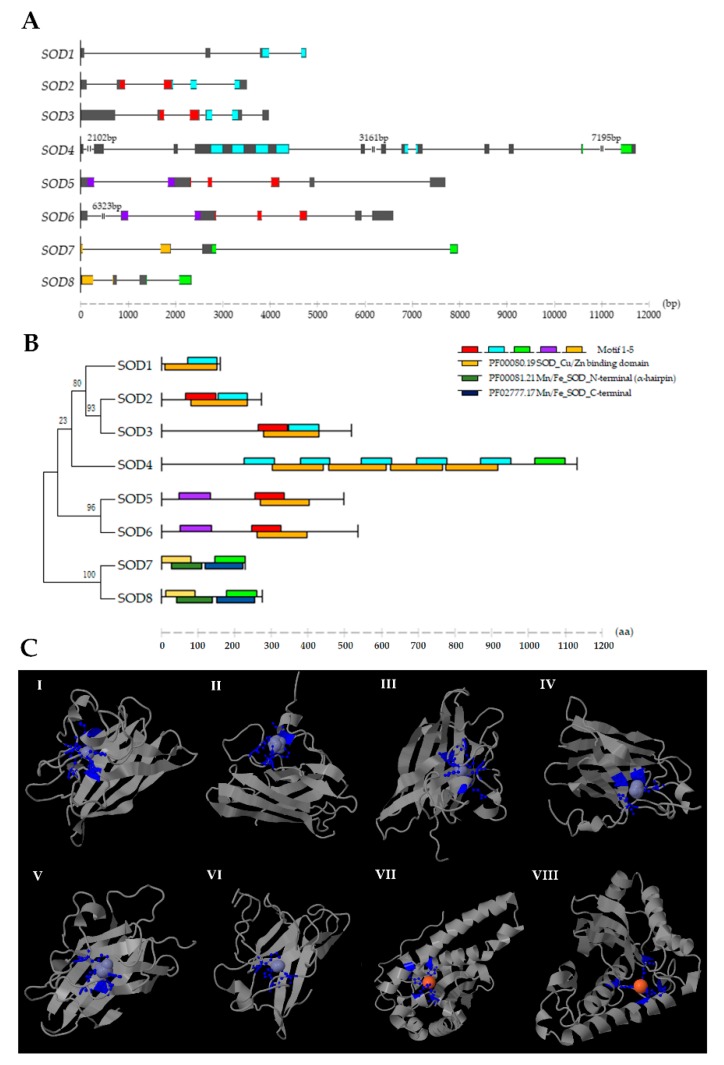
Exon/intron organization patterns of *SOD* genes in *C. farreri* as well as the conserved domain/motif and 3D structure analysis of the encoded proteins. (**A**) Schematic representation of the exon/intron structures of *SOD* genes. Each exon is represented in a grey box, and the locations of the nucleotide sequences which encode SOD motifs are indicated by colored boxes. The length of *SOD* genes can be estimated using the scale at the bottom. (**B**) Conserved domains and motifs of *C. farreri* SODs according to their predicted regions (Table S2), respectively, are present on the right side. The length of the protein and motif can be estimated using the scale at the bottom. Phylogenetic tree of *C. farreri* SODs is present on the left side. (**C**) Predicted 3D structures and binding sites of *C. farreri* SODs. I-VI: 3D structures of Cu/Zn-SODs (SOD1-6). VII, VIII: 3D structures of Mn-SOD proteins.

Various exon/intron organization patterns of *SOD* genes are presented in *C. farreri* (Figure 1A), and the number of introns were identified to range between three and nine. According to sequence analysis, the ORF lengths of *SOD* genes in *C. farreri* were from 462 to 3348 bp, and the encoded proteins containing 153 to 1115 amino acids. Most of the SOD proteins were predicted to be stable (instability index ≤40), and all the SODs were found to be hydrophilic based on a grand average of hydropathicity (GRAVY) analysis (the aliphatic index ranged from 44.73 to 88.45) (Table 1). The *C. farreri* Mn-SODs were found to be acidic, whereas the Cu/Zn-SODs showed variable isoelectric point (*pI*) values, with two members (SOD3 and SOD4) being basic in character.

For all Cu/Zn-SODs in *C. farreri*, Motif 1 or 2 which contained conserved copper ligands and the site involved in a disulfide bond could be detected (Figure 1B and Table 2), and they possess the topological signature of β-barrels with ligand clusters localized on the exterior (Figure 1C). The sub-cellular predictions showed that SOD1 was localized in the cytoplasm; SOD2, SOD3, and SOD4 were mainly transported to the extracellular milieu; and first time in animals, we found two specific nucleus localized Cu/Zn-SODs (SOD5 and SOD6) in *C. farreri*. This finding was consistent with the results from the motif analysis that revealed a motif 4 which was classified as a specific ZapB domain (E-value: 1.2e-06) with a nuclear localization signal (score: 10) present in SOD5 and SOD6, and this ZapB domain has been reported to contribute to forming a coiled-coil structure and being involved in cell division [35] (Figure 1B). Notably, an unexpected motif 3 which contained manganese/iron ligands and showed conserved “D-x-[WF]-E-H-[STA]-[FY] (2)” Mn/Fe-SOD signature was found to be present at the C terminus of the “extremely long” Cu/Zn-SOD4 (Table 2). Meantime, a Fe ligand-bound Cys283 was also detected in SOD4 (Table S1), indicating that SOD4 might be a novel combined or transient SOD type with a complex catalytic metal ion-binding activity in *C. farreri*. In addition, SOD4 caught our notice due to its quadruple SOD_Cu domains, which were not found or reported in animals. We further retrieved SOD proteins from several mollusk species, and Cu/Zn-SODs with triple/quadruple SOD_Cu domains were also found in *B. glabrata* (XP_013062920.1), *C. gigas* (XP_019923318.1; XP_011414606.1), *Pinctada fucata* (ALK82329.1), and *Lottia gigantea* (V4AP91), which is suggestive of a mollusk-specific SOD type which originated from the common ancestor of these animals.

Mn-SODs in *C. farreri* possess both Motif 3 and 5, and SOD7 exhibited mitochondrial localization, while SOD8, for the first time in bivalves, was a SOD demonstrated to be a specific cytosolic type. Similar to bay scallop [36], the polypeptide chains of *C. farreri* Mn-SODs are divided into N-terminal helices and a C-terminal α/β domain, with the active metal ligand in the interior. The metal ion of Mn-SODs in *C. farreri* was found to be coordinated in a strained trigonal bipyramidal geometry by four amino acid side chains: His52-His100-Asp185-His189 and His61-His125-Asp214-His218 in SOD7 and SOD8, respectively. In the present study, all 3D models were validated by Ramachandran plot analysis (Figure S1), and the results showed that residues in the favored region ranged from 87.0% to 98.5%, and less than 3.8% were found in the outlier region, indicating fairly good quality (Table S3).

### 2.2. Phylogenetic Analysis of SODs

**Figure 2:**
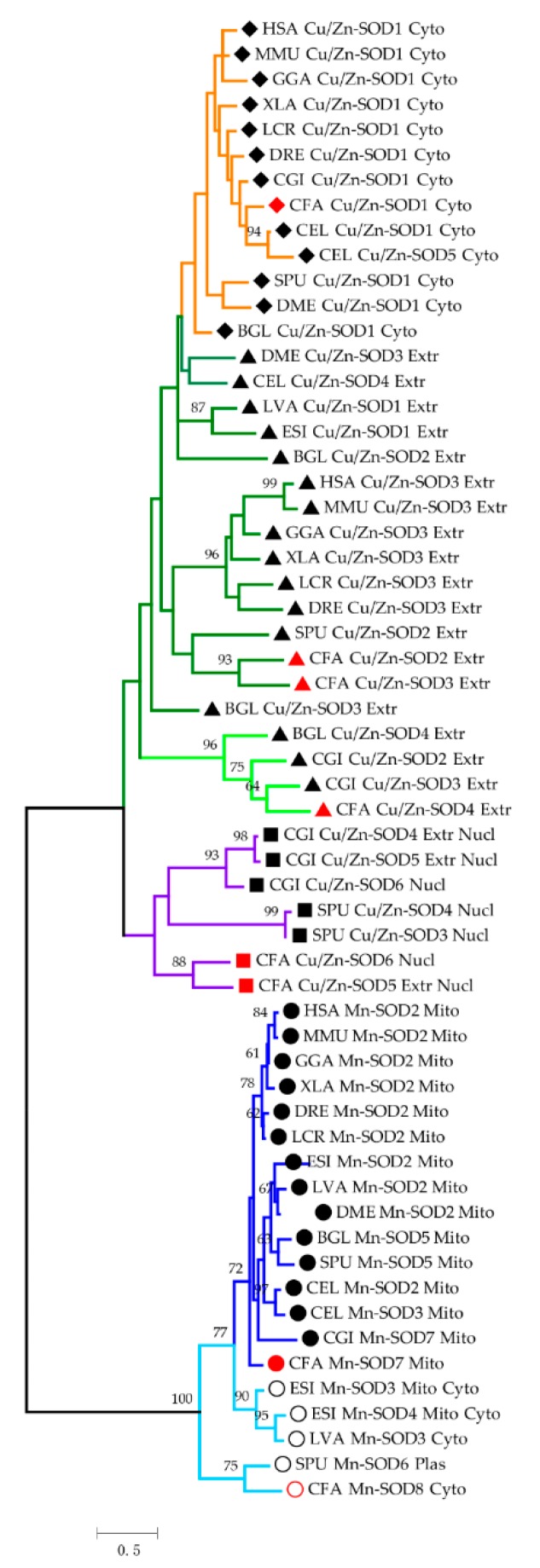
Phylogenetic tree of SODs from selected organisms. The tree is drawn to scale, with branch lengths measured based on the number of substitutions per site. Different symbols were used to distinguish the sub-cellular prediction, and the cytosolic, extracellular, and nuclear Cu/Zn-SODs are represented by solid diamonds, triangles, and squares, respectively. For Mn-SODs, circles and hollow circles are used to respectively represent mitochondrial and cytosolic Mn-SODs. SODs of *C. farreri* are specifically highlighted by the reddish symbol. The SODs were classified into two major groups and five subfamilies, which are shown as orange, green, purple, light blue, and dark blue branches, respectively. HSA: *Homo sapiens*, MMU: *Mus musculus*, GGA: *Gallus gallus*, XLA: *Xenopus laevis*, DRE: *Danio rerio*, LCR: *Larimichthys crocea*, SPU: *Strongylocentrotus purpuratus*, BGL: *Biomphalaria glabrata*, CGI: *Crassostrea gigas*, LVA: *Litopenaeus vannamei*, ESI: *Eriocheir sinensis*, DME: *Drosophila melanogaster*, CEL: *Caenorhabditis elegans*, and CFA: *Chlamys farreri.*

Based on polygenetic analysis (Figure 2), distinct evolutionary paths for Mn- SODs and Cu/Zn-SODs with varying degrees of protein conservation were observed. In addition, SOD members with different subcellular locations diverged from each other at early stages of evolution, prior to the differentiation of invertebrates and vertebrates, suggesting the rapid sequence divergence of SODs. All mitochondrial Mn-SODs across 14 species were clustered together, and two branches formed by vertebrates and invertebrates could be tracked, indicating highly conserved protein structures and evolutionary lineages. Prior to mitochondrial Mn-SODs, the cytosolic Mn-SODs near the phylogenetic root were clustered together firstly, indicating that these two types of Mn-SODs diverged long ago.

All the Cu/Zn-SODs in eukaryotes formed a large clade and comprising three subgroups, highly consistent with the subcellular predictions. Obviously, the cytosolic Cu/Zn-SODs (indicated by an orange color) detected across all species were clustered together, except in *L. vannamei* and *E. sinensis* since they do not have cytosolic Cu/Zn-SOD in their genome. Aquatic crustaceans have been reported to usually lack cytosolic Cu/Zn-SODs, and the relatively ancient cytosolic Mn-SODs might be linked to the fluctuation in copper metabolism induced by the special copper-dependent oxygen carrier protein hemocyanin [37,38,39,40]. For extracellular Cu/Zn-SODs with single functional domain, a clear branch for vertebrate Cu/Zn-SOD3 was detected, with conserved vertebrate-specific residues being found (Figure S2). In this context, the branch represented in light green (96% bootstrap value) was restricted to mollusk-specific extracellular Cu/Zn-SODs, which is attributed to the multiple tandem SOD_Cu domains. In addition, the potential ancient nuclear Cu/Zn-SODs (purple color) were only found from the phyla Molluska (*C. farreri*, *C. gigas*) and Echinodermata (*S. purpuratus*). The complex phylogenetic relationship of Cu/Zn-SODs may be due to their flexible plastic N- and C-termini decorated with localization signal peptides, and Cu/Zn-SODs may have evolved independently multiple times after the divergence of different lineages [41,42], indicating the differential interspecific evolution rates as well as rapid intraspecific sequence divergence of these proteins.

### 2.3. Spatiotemporal Expression Profiles of Scallop SODs During Development and in Adult Organs/Tissues

**Figure 3:**
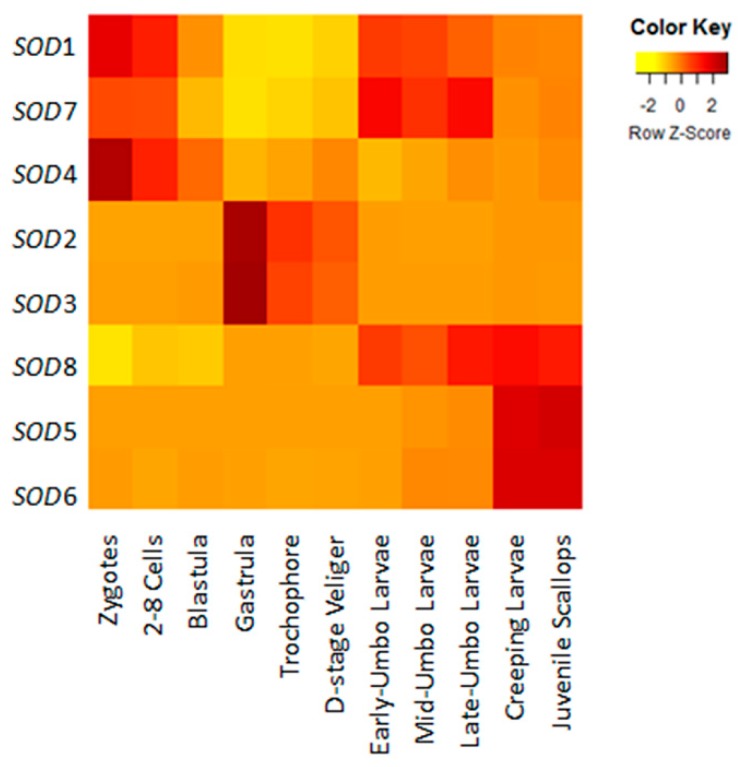
Heat map of *C. farreri SOD*s expression during development. Expression of eight *SOD*s from zygotes; 2–8 cells; blastula; gastrula; trochophore; D-stage veliger; early-, mid-, and late-term umbo larvae; creeping larvae; and juvenile scallops were tested. The reads per kilobase of exon model per million mapped reads (RPKMs) calculated from the RNA-seq data are shown as a heat map. The color varies from yellow to brown, representing the scale of the relative expression level.

During embryonic and larval development, the temporal activation of *SOD* genes and their expression patterns could be clearly distinguished in *C. farreri* (Figure 3). In multicellular stage, a set of *SOD* transcripts, including cytosolic *Cu/Zn-SOD1*, extracellular *Cu/Zn-SOD4,* and mitochondrial *Mn-SOD7*, were detected at the very beginning of fertilization and exhibited high expression until blastula formation, suggesting their maternal origin to play protective roles and to help maintain a redox balance during fertilization and cell cleavage. Afterwards, dominant expression was observed for extracellular *Cu/Zn-SOD2* and *Cu/Zn-SOD3* during gastrulation, from which more than 200-fold elevated mRNA level was detected, and their high expression was maintained until D-stage veliger formation. When get into umbo larvae development, the expression levels of *SOD1* and *SOD7* were respectively enhanced 3.3-and 12.2-fold again, together with a significant activation of cytosolic *Mn-SOD8*. Nevertheless, only *Mn-SOD8* could exhibit persistent high expression in creeping larvae as well as in juvenile scallops, during which the expression of nuclear *Cu/Zn-SOD5* and *Cu/Zn-SOD6* was remarkedly increased. The participation of *SOD*s in gastrulation and metamorphosis has been found by several lines of evidence, including in prawn [43], seabass [44], frog [45], fruit fly [46], and chicken [47], which may due to the elevation of oxygen consumption to meet the high demands of energy reserve utilization during organ initiation and structural remodelling. Studies in mouse embryos also found that, regardless of whether fertilization had occurred in vivo or in vitro, addition of SOD led to a protective effect against oxidative stress on both sperm viability and fertilized embryos [48]. Thus, the explicit temporal expression patterns of *SOD*s observed in the present study may suggest their important roles in key processes during development, indicating the indispensability of *SOD*s for organ/tissue initiation and maturation in *C. farreri*.

**Figure 4:**
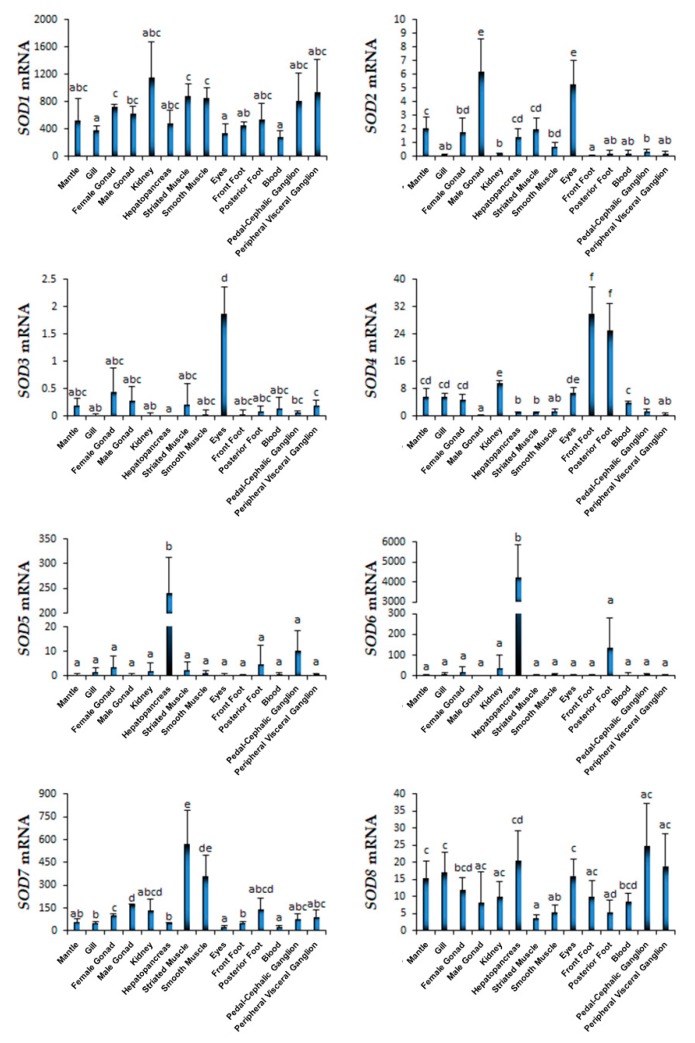
Expression of *C. farreri SOD*s in adult tissues/organs. The expression of eight *SOD*s in the mantle, gill, female gonad, male gonad, kidney, digestive gland, striated muscle, smooth muscle, eyes, front foot, posterior foot, blood, pedal-cephalic ganglion, and peripheral visceral ganglion was tested. The RPKMs calculated from the RNA-seq data are shown as a column diagram. Different letters indicate significant differences between groups (*p* < 0.05).

We further investigated the transcriptional profiles of *SOD* genes in 14 organs/tissues of adult scallop (Figure 4). Clearly, *SOD1* was the only *Cu/Zn-SOD* showed widespread expression in all the examined organs/tissues with significantly higher read per kilobase of exon model per million mapped reads (RPKM) values (*p* < 0.001) than most of the other *SOD*s; relatively high levels were detected in kidneys, muscles, and ganglions. Similar widespread tissue expression was observed for cytosolic *Mn-SOD8*, while the transcript amount was much lower than that of *SOD1*. Hepatopancreas and ganglions showed higher expression of *Mn-SOD8* than other organs/tissues. The expression of extracellular *Cu/Zn-SOD2* and *Cu/Zn-SOD3* was much lower than other *SOD* genes, with the male gonad exhibiting a high level of *SOD2* and the eye showing a high expression for both *SOD2* and *SOD3*. Similar results have been reported during investigation of mammal extracellular *SOD*s, which revealed the protective function of extracellular *SOD* in the corneal endothelium [49,50] and on the Sertoli/germ cell surface in testicles [51]. Of note, the dominant transcript mRNA of extracellular *Cu/Zn-SOD4* was found in the foot/byssus of *C. farreri*. Similarly, the same extracellular *Cu/Zn-SOD* (ALK82329.1) with a quadruple SOD_Cu domain was identified from the distal thread region of the byssus in *P. fucata*, and the researchers proposed that extracellular *SOD* could be required for prevention of the degradation of threads within the oxidative seawater environment [52]. The tissue expression of nuclear *Cu/Zn-SOD5* and *Cu/Zn-SOD6* caught our attention due to their outstanding hepatopancreas-specific expression, with more than 900-fold enhancement compared with other tissues, indicating a specialized tissue-specific function. Interestingly, the mitochondrial *Mn-SOD7* in *C. farreri* showed rather high levels in the striated muscle and smooth muscle, the primary organs associated with energy and mobility in scallops [25], implying this gene play important roles against oxidative stress in muscle. Previous study of mice found that conditional knockout of *Mn-SOD* targeted to type IIB skeletal muscle fibers not only can lead to oxidative stress enhance, but also is sufficient to reduce contractile muscle force and alter aerobic exercise capacity [53].

### 2.4. Diversified Expression Regulation of SODs in Response to PSTs Producers

Previous studies have documented that SOD activities were induced in bivalves when exposed to toxic algae [31,32], but the expression of underlying *SOD* genes has not been revealed. Our previous study indicated that the hepatopancreas and kidney in *C. farreri* are both toxin-rich organs containing the highest concentrations of PSTs [25]. To gain a deeper understanding of the defensive mechanism of bivalve *SOD* genes in response to PST-producing algae challenge, expression regulation of the *SOD* gene family in these two vulnerable organs of *C. farreri* challenged with PST-producing algae *A. minutum* (strain AM-1) and *A. catenella* (strain ACDH) were analyzed.

**Figure 5:**
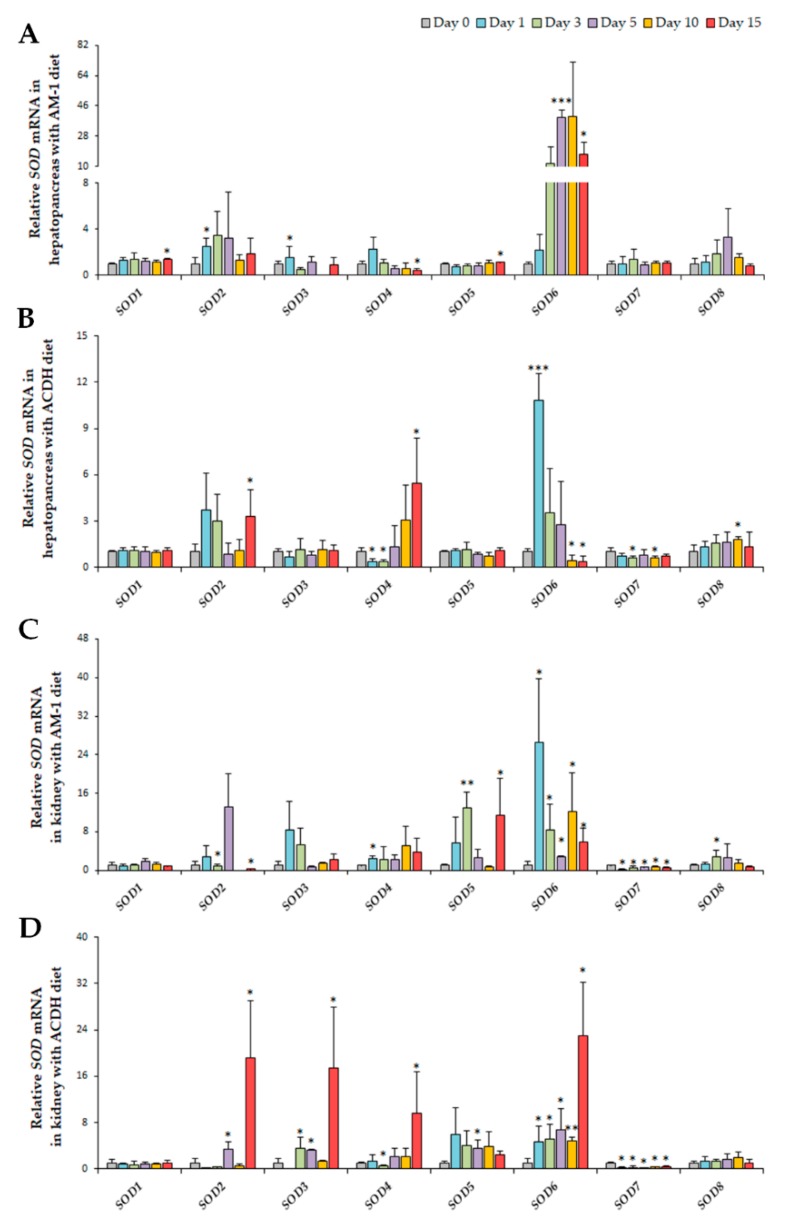
Acute (≤ day 3) and chronic (≥ day 5) responses of *SOD*s in *C. farreri* hepatopancreas (**A**,**B**) and kidney (**C**,**D**) after exposure to the toxic dinoflagellate *A. minutum* (**A**,**C**) *and A. catenella* (**B**,**D**). The relative variation tendencies after toxic diet exposure and the relative fold changes compared with control group for each test point are shown as a bar chart (significance: *** *p* < 0.001; ** *p* < 0.01; * *p* < 0.05).

In scallop hepatopancreas which is the main organ for PST uptake from algae, all the six *Cu/Zn-SOD*s showed significant alterations after *A. minutum* exposure, with *SOD1*, *2*, *3*, *5*, and *6* being upregulated and *SOD4* being downregulated, while no significant change was detected in *Mn-SOD*s (Figure 5A). Notably, the most dramatic upregulation was observed in *SOD6*, and the fold changes reached 39.01 and 17.10, respectively, on days 5 and 15. Chronic induction was also observed in *SOD1* and *SOD5* at 15 days post exposure, while *SOD2* and *SOD3* showed acute up-regulation on day 1. For *SOD4*, chronic suppression was detected on day 15. After exposure to *A. catenella*, up-regulation was observed in *SOD2*, *4*, *6*, and *8*, while *SOD7* was down-regulated. As shown in Figure 5B, significant acute induction of *SOD6* (10.82-fold) was observed on day 1, and chronic induction was found in *SOD2* (day 15), *SOD4* (day 15), and *SOD8* (day 10). Acute and chronic suppression of *SOD4* (on day 1 and 3) and *SOD6* (on day 10 and 15) was also observed, respectively. In addition, acute and chronic suppression of *SOD7* was detected on day 3 and day 10, respectively. Taken together, *Cu/Zn-SOD6* showed the most dramatic induction for both *A. minutum* and *A. catenella* exposure, implying that *SOD6* plays an important role in the antioxidant protection during PST accumulation in the hepatopancreas and may be a promising hepatopancreatic indicator gene during toxic dinoflagellate challenge in *C. farreri*. Furthermore, as the two algae contained different PST members, with *A. minutum* mainly synthesizing GTX1-4 and *A. catenella* synthesizing C1-2, the chronic response of *SOD6* for *A. minutum* exposure and its acute response for *A. catenella* exposure suggests that the activation of *SOD6* for antioxidant defense is dependent on the species or toxicity of the PSTs accumulated. Similar phenomena were also observed for *SOD4*, from which we could detect its chronically suppressed expression after *A. minutum* exposure, while acute suppression followed by chronic stimulation of *SOD4* was observed after *A. catenella* exposure. These findings all indicate dinoflagellate-dependent responses of *SOD* members in hepatopancreas. Meanwhile, we further found that expression of *SOD3* and *SOD5* was negatively correlated (*p* < 0.05) for both *A. minutum* and *A. catenella* exposure (Figure S3A,B), suggesting their complementary or substitutionary function in scallop hepatopancreas to cope with PSTs producing algae exposure.

In the kidney, where the ingested PSTs are transformed to more toxic analogs [25], all the *SOD*s except *SOD1* showed significant alteration at least at one time point (Figure 5C,D). Among *Cu/Zn-SOD*s, after *A. minutum* exposure, rapid elevation of *SOD4* (2.47-fold), *SOD5* (12.89-fold), *SOD6* (26.58-fold), and *SOD8* (2.81-fold) expression was observed, and significant activation was maintained until day 15 for *SOD6*. In addition, acute suppression of *SOD2* and *SOD7* was observed, and *SOD7* was supressed at all the time points examined. After *A. catenella* exposure, all the up-regulated members were from *Cu/Zn-SOD*s, including *SOD2*, *3*, *4*, *5*, and *6*, and except for *SOD5*, the highest fold change of these genes was present at 15 days after exposure. Like the regulation pattern during *A. minutum* exposure, *SOD6* was up-regulated and *SOD7* was down-regulated at all the sampling time points after the *A. catenella* challenge. Meanwhile, in the kidney, *SOD6* showed the highest fold change among all the *SOD*s after the challenge of both algae, similar to the results in hepatopancreas. In addition, highly positive correlation was observed between the expression of *SOD3* and *SOD6* (*p* < 0.01) in scallop kidney, for both *A. minutum* and *A. catenella* exposure, with the coefficients of 0.67 and 0.74, respectively (Figure S3C,D), indicating their co-regulation in response to PSTs producing algae challenge in kidney.

Overall, in scallops after exposure to different toxic algae, *SOD* up-regulation mainly occurred in the expanded *Cu/Zn-SOD* group, and *SOD6* could be the promising indicator gene due to its highest fold change among all the *SOD*s and being up-regulated under all PST-producer challenge scenarios. These findings may indicate the importance of *Cu/Zn-SODs*, especially *SOD6* in protecting scallop from the stress of PSTs. In addition, diversified responsive patterns of *SOD* genes were detected in two toxin-rich organs after a *A. minutum* or *A. catenella* challenge according to the present data, with some members being up-regulated, some down-regulated, and some other members showing different regulation directions at different sampling times, depending on the examined organs and ingested algae. The diverse regulation pattern of *SOD*s provides important information for understanding the mechanism of SOD enzymes in protecting scallop organs/tissues against PSTs accumulation, as the enzyme activities were determined by the expression regulation of all the *SOD* genes. Our results suggest the diverse function of scallop *SOD*s during development and in response to PST-producing algae challenge, and the expansion of *Cu/Zn-SOD*s might be implicated in the adaptive evolution of scallop or bivalve with respect to antioxidant defense against the ingested toxic algae.

## 3. Conclusions

Through genome and transcriptome screening, we performed the first systematic analysis of *SOD* gene family in bivalves, and found the expansion of *Cu/Zn-SOD* in scallop. The considerable structural diversity, distinct sub-cellular localization, conserved domains and motifs, as well as various spatiotemporal expression patterns during development and in adult tissues/organs were also revealed. After exposure to two different toxic *Alexandrium spp*., scallop *SOD*s exhibited different regulation patterns depending on the algae strain ingested and scallop organ analyzed, with up-regulation mainly occurring in the expanded *Cu/Zn-SOD* group. In addition, *SOD6* was the only member that was dramatically up-regulated in both hepatopancreas and kidney among all the *SOD*s, implying the importance of *Cu/Zn-SOD*, especially *SOD6* in protecting scallop from the stress of PSTs.

## 4. Materials and Methods

### 4.1. Genome-Wide Identification and Sequence Analysis of SOD Genes in C. farreri

The *C. farreri* genomic and transcriptomic files were used for gene prediction and sequence analysis [25]. *De novo* predicted *SOD* genes were aligned to the genome to support the mRNA’s existence. The retrieved *SOD*s candidate sequences were translated through ORF finder (http://www.ncbi.nlm.nih.gov/gorf/gorf.html). Then, the predicted *C. farreri* SOD proteins were aligned to KEGG (http://www.kegg.jp/), UniProt (http://www.uniprot.org/), and the NCBI non-redundant protein sequence database with BLASTP (E-Value set: 1.00 × 10^−5^). All candidate SODs with a significant BLAST hit (*E*-value ≤ 1.00 × 10^−10^) were collected, and the presence of SOD domain was further verified by HMMER search (http://www.ebi.ac.uk/Tools/hmmer/search/phmmer) and SMART (http://smart.embl-heidelberg.de/) tools. To find conserved motifs in *C. farreri* SOD proteins, “Multiple EM for Motif Elicitation” (MEME) version 3.5.4 (http://meme-suite.org/tools/meme) was used with the following parameter settings: number of repetitions, any; maximum number of motifs, 6; optimum width of motif, 80–150 aa [54].

Characterization of the *C. farreri* SOD proteins, including the length (aa), molecular weight (kDa), isoelectric point (*pI*), instability index, aliphatic index, and grand average of hydropathicity (GRAVY) values, were performed using the ProtParam tool (http://www.expasy.org/tools/protparam.html), and the subcellular localization prediction of the SOD proteins was analyzed through CELLO v.2.5 (http://cello.life.nctu.edu.tw/) [55], Euk-mPLoc 2.0 (http://www.csbio.sjtu.edu.cn/bioinf/euk-multi-2/) [56] as well as WoLF PSORT II (https://www.genscript.com/wolf-psort.html) [57]. The nuclear localization signal was predicted by the cNLS Mapper program (http://nls-mapper.iab.keio.ac.jp/cgi-bin/NLS_Mapper_form.cgi) [58]. The signal peptide for mitochondrial translocation was predicted by using MitoFates (http://mitf.cbrc.jp/MitoFates/cgi-bin/top.cgi) [59]. The structures of SOD proteins were predicted using the Phyre server (http://www.sbg.bio.ic.ac.uk/phyre) [60]. We performed further ligand clustering and binding site prediction for *C. farreri* SODs using the 3D LigandSite server (http://www.sbg.bio.ic.ac.uk/3dligandsite/) [61] and their conserved disulfide bonds with cysteine residues were predicted by the DiANNA 1.1 server (http://clavius.bc.edu/~clotelab/DiANNA/) [62]. Structural evaluation was assessed using Ramachandran plot analysis (http://mordred.bioc.cam.ac.uk/~rapper/rampage.php) [63].

### 4.2. Multiple Alignment and Phylogenetic Analysis

Phylogenetic tree of *C. farreri* SODs was constructed with MEGA 7.0 by using the maximum likelihood method with bootstrap values as 1000 replicates. The protein sequences encoded by *SOD* genes identified from *C. farreri* and other selected species, including human (*Homo sapiens*), mouse (*Mus musculus*), chicken (*Gallus gallus*), frog (*Xenopus laevis*), zebrafish (*Danio rerio*), large yellow croaker (*Larimichthys crocea*), sea urchin (*Strongylocentrotus purpuratus*), freshwater snail (*Biomphalaria glabrata*), Pacific oyster (*Crassostrea gigas*), shrimp (*Litopenaeus vannamei*), crab (*Eriocheir sinensis*), fruit fly (*Drosophila melanogaster*), and nematode (*Caenorhabditis elegans*), were used to perform phylogenetic analysis with the maximum-likelihood method [64]. The SOD protein sequences from these species were retrieved from UniProt (http://www.uniprot.org/), Ensembl (http://asia.ensembl.org/index.html) and NCBI (http://www.ncbi.nlm.nih.gov/) databases, and the accession numbers, SOD type, as well as sub-cellular prediction are summarized in Table S4. Multiple protein sequences alignments were conducted using the Clustal W program, and the Jones-Taylor-Thornton (JTT) model with a bootstrap analysis of 1000 replicates was selected as the best fit model by MEGA 7 [65].

### 4.3. Expression Profiles of SOD Genes During C. farreri Development and in Adult Organs/Tissues

All experiments on scallops were conducted following institutional and national guidelines. The expression profiles of *SOD* genes in scallop developmental stages and adult organs/tissues were analyzed based on the RNA-seq data [25,66]. Briefly, embryos/larvae and adults of *C. farreri* were collected from the hatchery of Xunshan Group Co., Ltd. (Shandong, China). Embryo/larva samples were harvested at different developmental stages, including zygotes; 2–8 cells; blastula; gastrula; trochophore; D-stage veliger; early-, mid-, and late-term umbo larvae; creeping larvae and juvenile scallops, which were frozen in liquid nitrogen and stored at −80 °C until RNA extraction. The adults were acclimated in filtered and aerated seawater at 12–13 °C for one week. Then, 14 soft tissues (mantle, gill, female gonad, male gonad, kidney, digestive gland, striated muscle, smooth muscle, eyes, front foot, posterior foot, blood, pedal-cephalic ganglion, and peripheral visceral ganglion) were dissected and were frozen in liquid nitrogen and stored at −80 °C until RNA extraction.

Total mRNA was extracted using the conventional guanidinium isothiocyanate method. In brief, tissues were homogenized in lysis buffer (4 M guanidium thiocyanide, 25 mM sodium citrate pH 7.0, 0.5% sarcosyl, 0.1 M 2-mercaptoethanol) followed by phenol/chloroform extraction. Total RNA was precipitated in ethanol, washed, and dissolved in DEPC water. The RNA-seq libraries were constructed using the NEB Next mRNA Library Prep Kit following the manufacturer’s instructions and were subjected to PE125 sequencing on the Illumina HiSeq 2000 platform. The generated RNA-seq reads were mapped to the C. farreri genome using Tophat (ver 2.0.9), and the expression of all *SOD* genes was normalized and represented in the form of reads per kb of exon model per million mapped reads (RPKMs).

### 4.4. Expression Response of SOD Genes to A. minutum and A. catenella Exposure

Previously, adult *C. farreri* was challenged with the PST-producing dinoflagellates *A. minutum* (strain AM-1) and *A. catenella* (strain ACDH) via feeding prior to sample collection [66]. No other noxious metabolites were previously reported in these two strains, but the main PST analogs in AM-1 and ACDH were gonyautoxins (GTX1-4) and N-sulfocarbamoyl-gonyautoxins (C-1,2), respectively [25,66,67]. The dinoflagellate cultures were grown in L1 medium at 17 °C/25 °C with a light: dark cycle of 12:12 h and were collected during the exponential growth phase at a cell density approaching 5 × 10^4^ cells mL^−1^ [68,69]. Microalgal ration was provided once each day, with a final cell density of 2.5 × 10^3^ cells mL^−1^ during the feeding experiments. Three hours after feeding, three scallops were sampled at 0 days (control), 1 day, 3 days (acute response), 5, 10, and 15 days (chronic response). In addition, the hepatopancreas as well as kidneys from each scallop were harvested, washed with sterile seawater, and frozen at −80 °C for subsequent RNA extraction. RNA-seq data generated from the sampled scallop tissue were used to examine the expression profiles of *SOD* genes. Barcodes were used to discriminate the sequencing reads from different sampled individuals. The expression estimation of all *SOD* genes was normalized and represented in the form of RPKM. The fold changes in *SOD* expression at each test point were calculated and normalized to the data from the control group. Significant differences between the test and control groups were determined using an independent-samples T-Test (*p* < 0.05, n = 3).

## Figures and Tables

**Table 1:** Characterization of *Chlamys farreri SOD* genes and the coding proteins.

Gene Characteristics				Protein Characteristics						Sub-Cellular Localization Prediction			
Gene Name	NCBI ID	Genomic Position	ORF (bp)	Length (aa)	MW (kDa)	*pI*	Instability Index	Aliphatic Index	GRAVY	CELLO v.2.5	Euk-mPLoc 2.0	WoLF PSORT II	Final
*Cu/Zn-SOD1*	MK374365	50981.1: 5129-13060(+)	462	153	15615.35	6.1	7.98	75.16	−0.292	Cyto	Cyto	Cyto	Cyto
*Cu/Zn-SOD2*	MK374366	9607.44: 811894-816438(+)	855	284	30094.36	5.33	32.34	67.89	−0.341	Extr	Chloro	Extr	Extr
*Cu/Zn-SOD3*	MK374367	11157.31: 577292-581362(−)	1533	510	54202.24	9.2	34.37	44.73	−0.712	Extr	Extr	Extr	Extr
*Cu/Zn-SOD4*	MK374368	39551.24: 361068-385243(−)	3348	1115	120753.05	8.82	37.55	76.36	−0.254	Extr	Extr	Nucl	Extr
*Cu/Zn-SOD5*	MK374369	62345.9: 206833-214902(−)	1455	484	55240.85	5.57	35.29	58.99	−1.26	Nucl	Extr	Nucl	Nucl
*Cu/Zn-SOD6*	MK374370	10699.65: 1421918-1435004(+)	1572	523	58628.12	5.58	36.05	53.31	−1.283	Nucl	Chloro	Nucl	Nucl
*Mn-SOD7*	MK374371	62487.8: 111489-119434(+)	681	226	25020.53	6.44	38.82	88.45	−0.16	Mito	Mito	Mito	Mito
*Mn-SOD8*	MK374372	20055.26: 792736-795083(+)	789	262	30332.28	4.94	41.32 (un)	87.06	−0.346	Cyto	Chloro	Cyto	Cyto

Cyto: Cytoplasm Extr: Extracellular Nucl: Nuclear Mito: Mitochondrion Chloro: Chloroplast.

**Table 2:** Two best match motif alignment of *C. farreri* SOD proteins identified by MEME tools. The Copper/Zinc superoxide dismutase signatures “[GA]-[IMFAT]-H-[LIVF]-H-{S}-x-[GP]-[SDG]-x-[STAGDE]” (SOD_CU_ZN_1, PS00087) and “G-[GNHD]-[SGA]-[GR]-x-R-x-[SGAWRV]-C-x(2)-[IV]” (SOD_CU_ZN_2, PS00332) are underlined, from which the copper ligands and disulfide binding site are in red and yellow box, respectively. Besides, the consensus pattern of manganese superoxide dismutase “D-x-[WF]-E-H-[STA]-[FY] (2)” is double-underlined and manganese/iron ligands are in green box. The predicted monopartite Nuclear localization signal are in blue color (Score = 10).

Motif	Width (aa)	Consensus Sequence	Pfam Domain
1	80	EHEL[HY]A[HY]CECM[PF]N[KM][TQ]TPDIA[GY]KI[DR][GF]H[QI]ELTGEPHEDEVSIVYNL[PR]NLKPDTEHGIHIHEYGD[VM]GRCC[DY]SLGPHYNPTHKS	sod_Cu/Zn PF00080.19
2	80	R[DN]E[GM][GY]E[LE]RHYGDLGN[VM]RQDGKGVV[KM][TW]D[IF]VDKLLPL[SR]GP[TR]SV[LI]GRS[VM]VIHYD[NR]DD[LM]G[KR]GGN[VA][VM]S[LY][TQ]TGNAGT[RP]LAC[AC]VIAR	sod_Cu/Zn PF00080.19
3	80	G[GC]WGWLSRNPIS[NK]RPLVASKPT[NY]RPLQPT[DA][GE]LQPIFGIDVWEHAYY[LI][QK]YKNIRPKYVK[DR]WWNIVNW[DR][GK]V[VA]QF[GD]HW[AW][KY][GR]PC	Sod_Mn/Fe_C PF02777.17
4	85	CCCFAIPEFDPKPENM[DK][LM]KWFGALVALALLNPN[PE]G[DK][GQ]RRKRQRRSTAVVDEIAELKAKVEKLEEQVNKLEEKDLGVHIHIGND[HY]H	ZapB PF06005.11
5	81	WLLAFCYFIKVMPKKM[GP][AY]E[GF]IFDQRLN[HY]VLP[DK]LPYDFKDLEPFIDEEIMRIH[HY]L[GK]HHAAYVKK[LM]NIAEEKWAEDMEVKNV[NM]	Sod_Mn/Fe_N PF00081.21

## Supplementary Materials

The following are available online at https://www.mdpi.com/1660-3397/17/12/700/s1, Figure S1: Conserved motif logos as well as Ramachandran plots of *C. farreri* SODs, Figure S2: Amino acid sequence alignments of extracellular SODs, Figure S3: The Correlation Matrix Plots for expression of *SOD*s on *A. minutum* and *A. catenella* diet in hepatopancreas and kidney of *C. farreri*, Table S1: Conserved disulfide bonds with cysteine residues prediction of Cu/Zn-SODs in *C. farreri*, Table S2: The location and E-value for predicted Pfam domains as well as Motif arrays of *C. farreri* SODs, Table S3: The Ramachandran plot analysis of SOD proteins in *C. farreri*, Table S4: Species accession numbers of SOD used for phylogenetic analysis.
