# Supplementary material for: Genome-Wide Identification and Characterization of SODs in Zhikong Scallop Reveals Gene Expansion and Regulation Divergence after Toxic Dinoflagellate Exposure
Source: Mar Drugs. 2019 Dec 12;17(12):700. doi: 10.3390/md17120700 (PMC6949909; doi:10.3390/md17120700)
Supplement: Supplementary file 1 [file marinedrugs-17-00700-s001.pdf]

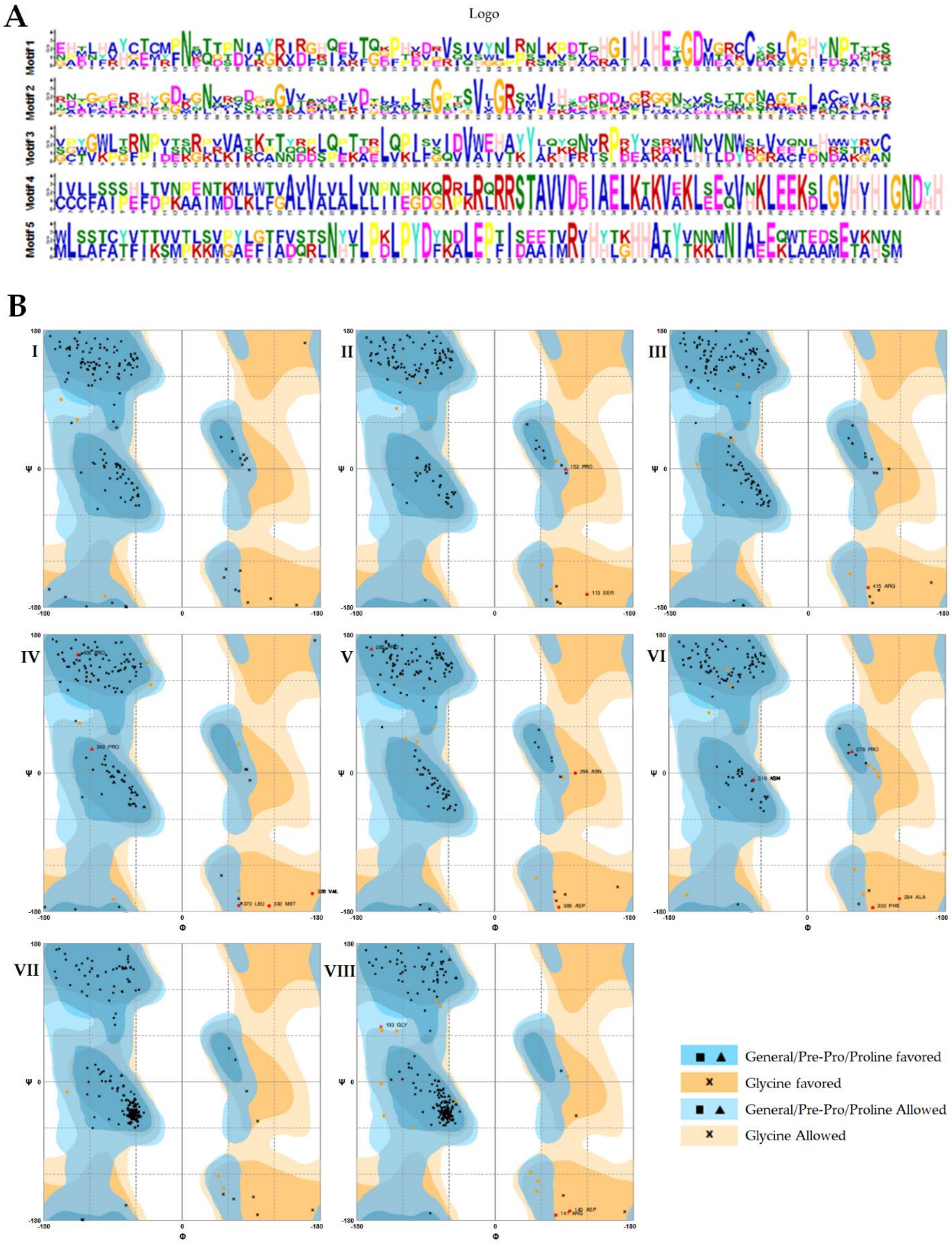

Figure S1. Conserved motif logos as well as Ramachandran plots of *C. farreri* SODs. **A.** Conserved motif logos in the SOD proteins of *C. farreri*. **B** Ramachandran plots of *C. farreri* SODs. Energetically allowed regions for backbone dihedral angles ψ against φ of amino acid residues in structures of CFA-SOD proteins (SOD1-8) were visualized in **I-**

VIII respectively. As indicated at the bottom right of the figure, contours for favored and for allowed conformational regions for the general case were shown in deep and light blue color, and all amino acids except Gly, Pro, and pre-Pro were marked as squares while pre-Pro/Proline were showed as triangles. For Glycine that was labelled by forks, the favored and allowed regions were separated by deep and light orange. The residues in the outlier region were high lightened by red color.

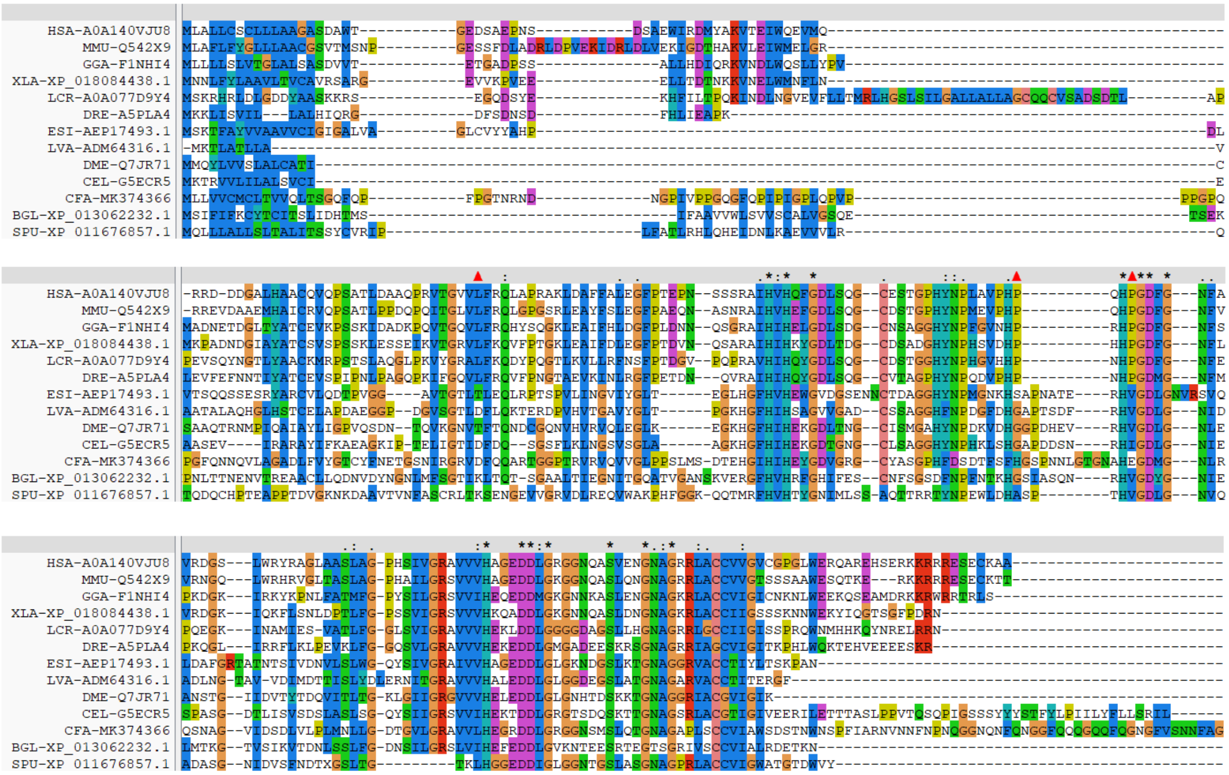

Figure S2. Amino acid sequence alignments of extracellular SODs. A line above the alignment is used to mark strongly conserved positions. Three characters ("*", ":" and ".") are used: "*" indicates positions which have a single, fully conserved residue. ":" indicates that one of the following 'strong' groups is fully conserved. "." indicates that one of the following 'weaker' groups is fully conserved. Solid triangle with reddish color represents the conserved vertebrate residues within the SOD_Cu domain.

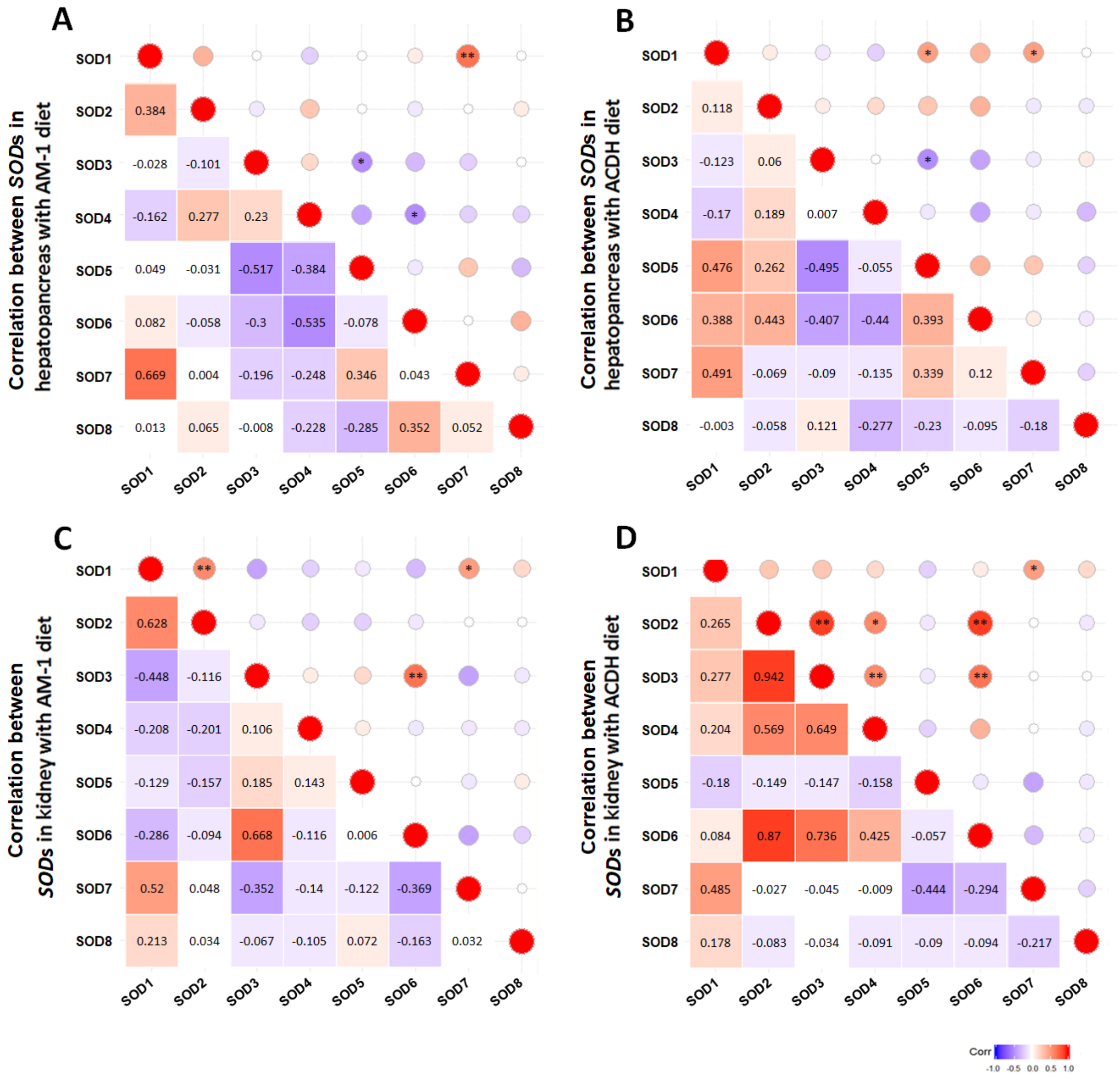

Figure S3. (A-D) The Correlation Matrix Plots for expression of *SODs* on *A. minutum* and *A. catenella* diet in hepatopancreas and kidney of *C. farreri*. The upper-triangle indicates the significance (***P<0.001; **P<0.01; *P<0.05), and the lower-triangle shows the correlation coefficient.

Table S1. Conserved disulfide bonds with cysteine residues prediction of Cu/Zn-SODs in *C. farreri*.

Genes	Cysteine Class prediction			Weighted matching disulfide bonds with high score			Connectivity
	Half-cystine	Free cystine	Ligand-bound	Cysteine position	Bond Sequence	Score	
SOD1	Cys56	Cys145	Cys7 (Zn)	Cys7/Cys56	SVTAVCVLKGD-DNTNGCTSAGA	0.99915	1-2
SOD2	-	Cys6, Cys8, Cys77, Cys134, Cys227, Cys228	-	Cys77/Cys134	FVYGTCYFNET - DVGRGCYASGP	0.97537	1-5, 2-6, 3-4
SOD3	Cys272	Cys15, Cys420, Cys421	Cys329 (Zn)	Cys15/Cys272	IYLYVCVVLIL - YRYATCRFNST	0.99422	1-2, 3-5
SOD4	Cys434, Cys601, Cys844, Cys905	Cys12, Cys80, Cys128, Cys203, Cys220, Cys228, Cys352, Cys513, Cys535, Cys543, Cys669, Cys748, Cys824, Cys852, Cys958, Cys998, Cys1080, Cys1089, Cys1090, Cys1104	Cys283 (Fe)	Cys80/Cys220	DVAGRCNQLGQ - STGTGCSPQVQ	0.99295	1-25, 2-5, 3-8, 6-11, 7-21, 9-10, 12-14, 13-16, 15-17, 18-20, 19-22, 23-24
				Cys128/Cys352	PNVLSCATLEA - PGQEICGQEYV	0.99952	
				Cys228/Cys535	QVQLNCKAGDL - NYAQQCNSANQ	0.99977	
				Cys283/Cys998	SVPLACAKIIK - PSPSVCGAAKG	0.99968	
				Cys434/Cys513	GSRWICATIES - DVSQRCSSVGG	0.99490	
				Cys543/Cys669	ANQLRCELGDL-PSTGSCSATSV	0.97587	
				Cys748/Cys844	GTRWKCGNIVE - TNYAECSPSNQ	0.99698	
				Cys852/Cys958	SNQLRCEAGDL - SDGPGCVEVDV	0.99525	
				Cys905/Cys1080	GPRYVCGDIMP - YRGRVCFGHRS	0.99759	
SOD5	-	Cys24, Cys50, Cys243, Cys260, Cys318, Cys319, Cys394, Cys395	-	Cys24/Cys50	QDNGQCHLLSH-SAINPCVLLAI	0.99736	1-2, 3-8, 4-7, 5-6
SOD6	-	Cys12, Cys31, Cys54, Cys55, Cys235, Cys251, Cys309, Cys310, Cys389	Cys390 (Zn)	Cys31/Cys251	VWWQECGQISK - TLHAHCEMMPN	0.99800	1-7, 2-6, 3-9, 4-8, 5-10
				Cys55/Cys310	FLIICCFSSSE - DMTECCNSLGG	0.99150	
				Cys235/Cys390	KLSGSCGHGTT - GPVACCIIGLS	0.99966	

Table S2. The location and E-value for predicted Pfam domains as well as Motif arrays of *C. farreri* SODs.

Proteins	Predicted Pfam domain				Predicted Motifs	
	Description	Number	Position	E-value	Description	E-value
SOD1	PF00080.19 SOD_Cu/Zn binding domain	1	9-148	8.60E-54	70-[2]-3	1.30E-48
SOD2	PF00080.19 SOD_Cu/Zn binding domain	1	80-230	9.10E-36	67-[1]-5-[2]-52	2.50E-98
SOD3	PF00080.19 SOD_Cu/Zn binding domain	1	275-423	3.00E-33	262-[1]-3-[2]-85	2.30E-105
SOD4	PF00080.19 SOD_Cu/Zn binding domain	4	298-437	4.20E-19	225-[2]-70-[2]-86-[2]-69-[2]-93-[2]-66-[3]-26	2.50E-78
			451-607	3.40E-17		
			617-759	1.10E-16		
			766-908	4.00E-17		
SOD5	PF00080.19 SOD_Cu/Zn binding domain	1	267-397	1.80E-33	49-[4]-118-[1]-152	5.30E-149
SOD6	PF00080.19 SOD_Cu/Zn binding domain	1	258-392	6.80E-27	52-[4]-106-[1]-200	3.30E-152
SOD7	PF00081.21 Mn/Fe_SOD_N-terminal (α-hairpin)	1	27-108	5.60E-36	[5]-65-[3]	5.00E-132
	PF02777.17 Mn/Fe_SOD_C-terminal	1	115-218	1.80E-40		
SOD8	PF00081.21 Mn/Fe_SOD_N-terminal (α-hairpin)	1	36-133	8.00E-26	9-[5]-85-[3]-7	8.40E-138
	PF02777.17 Mn/Fe_SOD_C-terminal	1	143-247	3.60E-36		

Table S3. The Ramachandran plot analysis of SOD proteins in *C. farreri*.

Proteins	Evaluation of residues				
	% in Favored region	Allowed region		Outlier region	
		% (Number)	Residues list	% (Number)	Residues list
SOD1	146/149 (98.0%)	3/149 (2.0%)	Asn54, Asp97, Arg114	0/149 (0.0%)	-
SOD2	126/134 (94.0%)	6/134 (4.5%)	Thr97, Gly128, Val130, Asp144, Val193, Pro224	2/134 (1.5%)	Ser113, Pro152
SOD3	147/154 (95.5%)	6/154 (3.9%)	Gly323, Ala327, Lys328, Thr404, Asn411, Pro417	1/154 (0.6%)	Arg415
SOD4	120/131 (91.6%)	6/131 (4.6%)	Val341, Cys352, His360, Ser365, Ser387, Arg419	5/131 (3.8)	Val322, Met330, Pro342, Leu370, Pro409
SOD5	131/139 (94.2%)	5/139 (3.6%)	Gly313, Arg317, Cys318, Asp340, Gln354	3/139 (2.2%)	Pro285, Asn298, Asp366
SOD6	107/123 (87.0%)	12/123 (9.8%)	Tyr264, His277, Pro279, Leu287, Gly304, Met306, Thr320, Ser327, Thr350, Leu365, Pro386, Gly393	4/123 (3.3%)	Pro276, Ala294, Asn318, Phe330
SOD7	193/196 (98.5)	3/196 (1.5%)	Asn168, Tyr191, Lys196	0/196 (0.0%)	-
SOD8	189/204 (92.6%)	12/204 (5.9%)	Ser35, Glu50, Phe52, Gly64, Trp80, Asn145, Leu172, Asn198, Glu206, Pro209, Tyr220, Gln225	3/204 (1.5%)	Gly103, Asp140, Arg141

Table S4: Species accession numbers of SOD used for phylogenetic analysis.

Species Name	Abbreviation	Latin name	Protein ID (Uniprot/Ensembl/NCBI)	SOD Type	Subcellular Prediction
Human	HSA	*Homo sapiens*	V9HWC9	Cu/Zn-SOD1	Cytosolic
Human	HSA	*Homo sapiens*	P04179	Mn-SOD2	Mitochondrial
Human	HSA	*Homo sapiens*	A0A140VJU8	Cu/Zn-SOD3	Extracellular
Mouse	MMU	*Mus musculus*	P08228	Cu/Zn-SOD1	Cytosolic
Mouse	MMU	*Mus musculus*	Q4FJX9	Mn-SOD2	Mitochondrial
Mouse	MMU	*Mus musculus*	Q542X9	Cu/Zn-SOD3	Extracellular
Chicken	GGA	*Gallus gallus*	P80566	Cu/Zn-SOD1	Cytosolic
Chicken	GGA	*Gallus gallus*	Q9DDJ1	Mn-SOD2	Mitochondrial
Chicken	GGA	*Gallus gallus*	F1NHI4	Cu/Zn-SOD3	Extracellular
Frog	XLA	*Xenopus laevis*	A0A1L8HCL3	Cu/Zn-SOD1	Cytosolic
Frog	XLA	*Xenopus laevis*	Q6UTE6	Mn-SOD2	Mitochondrial
Frog	XLA	*Xenopus laevis*	XP_018084438.1	Cu/Zn-SOD3	Extracellular
Zebrafish	DRE	*Danio rerio*	B2GRH9	Cu/Zn-SOD1	Cytosolic
Zebrafish	DRE	*Danio rerio*	Q6P980	Mn-SOD2	Mitochondrial
Zebrafish	DRE	*Danio rerio*	A5PLA4	Cu/Zn-SOD3	Extracellular
Large yellow croaker	LCR	*Larimichthys crocea*	A0A077DB40	Cu/Zn-SOD1	Cytosolic
Large yellow croaker	LCR	*Larimichthys crocea*	A0A077D814	Mn-SOD2	Mitochondrial
Large yellow croaker	LCR	*Larimichthys crocea*	A0A077D9Y4	Cu/Zn-SOD3	Extracellular
Sea urchin	SPU	*Strongylocentrotus purpuratus*	XP_784574.2	Cu/Zn-SOD1	Cytosolic
Sea urchin	SPU	*Strongylocentrotus purpuratus*	XP_011676857.1	Cu/Zn-SOD2	Extracellular
Sea urchin	SPU	*Strongylocentrotus purpuratus*	W4XNU0	Cu/Zn-SOD3	Nuclear
Sea urchin	SPU	*Strongylocentrotus purpuratus*	W4YE27	Cu/Zn-SOD4	Nuclear

Sea urchin	SPU	*Strongylocentrotus purpuratus*	XP_785278.3	Mn-SOD5	Mitochondrial
Sea urchin	SPU	*Strongylocentrotus purpuratus*	W4Z127	Mn-SOD6	PlasmaMembrane
Freshwater snail	BGL	*Biomphalaria glabrata*	XP_013070344.1	Cu/Zn-SOD1	Cytosolic
Freshwater snail	BGL	*Biomphalaria glabrata*	XP_013062232.1	Cu/Zn-SOD2	Extracellular
Freshwater snail	BGL	*Biomphalaria glabrata*	XP_013070220.1	Cu/Zn-SOD3	Extracellular
Freshwater snail	BGL	*Biomphalaria glabrata*	XP_013062920.1	Cu/Zn-SOD4	Extracellular
Freshwater snail	BGL	*Biomphalaria glabrata*	NP_001298192.1	Mn-SOD5	Mitochondrial
Pacific oyster	CGI	*Crassostrea gigas*	K1QDI4	Cu/Zn-SOD1	Cytosolic
Pacific oyster	CGI	*Crassostrea gigas*	XP_019923318.1	Cu/Zn-SOD2	Extracellular
Pacific oyster	CGI	*Crassostrea gigas*	XP_011414606.1	Cu/Zn-SOD3	Extracellular
Pacific oyster	CGI	*Crassostrea gigas*	K1QLW5	Cu/Zn-SOD4	Extracellular_Nuclear
Pacific oyster	CGI	*Crassostrea gigas*	K1RVZ4	Cu/Zn-SOD5	Extracellular_Nuclear
Pacific oyster	CGI	*Crassostrea gigas*	K1P1M6	Cu/Zn-SOD6	Nuclear
Pacific oyster	CGI	*Crassostrea gigas*	B1A4F6	Mn-SOD7	Mitochondrial
Shrimp	LVA	*Litopenaeus vannamei*	ADM64316.1	Cu/Zn-SOD1	Extracellular
Shrimp	LVA	*Litopenaeus vannamei*	AKM12646.1	Mn-SOD2	Mitochondrial
Shrimp	LVA	*Litopenaeus vannamei*	AAY89338.1	Mn-SOD3	Cytosolic
Crab	ESI	*Eriocheir sinensis*	AEP17493.1	Cu/Zn-SOD1	Extracellular
Crab	ESI	*Eriocheir sinensis*	AFN29184.1	Mn-SOD2	Mitochondrial
Crab	ESI	*Eriocheir sinensis*	ADF45346.1	Mn-SOD3	Mitochondrial_Cytosolic
Crab	ESI	*Eriocheir sinensis*	ACV41936.1	Mn-SOD4	Mitochondrial_Cytosolic
Fruit fly	DME	*Drosophila melanogaster*	P61851	Cu/Zn-SOD1	Cytosolic
Fruit fly	DME	*Drosophila melanogaster*	A0A0B4LGQ1	Mn-SOD2	Mitochondrial
Fruit fly	DME	*Drosophila melanogaster*	Q7JR71	Cu/Zn-SOD3	Extracellular
Nematode	CEL	*Caenorhabditis elegans*	P34697	Cu/Zn-SOD1	Cytosolic

Nematode	CEL	*Caenorhabditis elegans*	P31161	Mn-SOD2	Mitochondrial
Nematode	CEL	*Caenorhabditis elegans*	P41977	Mn-SOD3	Mitochondrial
Nematode	CEL	*Caenorhabditis elegans*	G5ECR5	Cu/Zn-SOD4	Extracellular
Nematode	CEL	*Caenorhabditis elegans*	Q27538	Cu/Zn-SOD5	Cytosolic
Zhikong scallop	CFA	*Chlamys farreri*	MK374365	Cu/Zn-SOD1	Cytosolic
Zhikong scallop	CFA	*Chlamys farreri*	MK374366	Cu/Zn-SOD2	Extracellular
Zhikong scallop	CFA	*Chlamys farreri*	MK374367	Cu/Zn-SOD3	Extracellular
Zhikong scallop	CFA	*Chlamys farreri*	MK374368	Cu/Zn-SOD4	Extracellular
Zhikong scallop	CFA	*Chlamys farreri*	MK374369	Cu/Zn-SOD5	Nuclear
Zhikong scallop	CFA	*Chlamys farreri*	MK374370	Cu/Zn-SOD6	Nuclear
Zhikong scallop	CFA	*Chlamys farreri*	MK374371	Mn-SOD7	Mitochondrial
Zhikong scallop	CFA	*Chlamys farreri*	MK374372	Mn-SOD8	Cytosolic
